# Video Abnormal Behavior Recognition and Trajectory Prediction Based on Lightweight Skeleton Feature Extraction

**DOI:** 10.3390/s24123711

**Published:** 2024-06-07

**Authors:** Ling Wang, Cong Ding, Yifan Zhang, Tie Hua Zhou, Wei Ding, Keun Ho Ryu, Kwang Woo Nam

**Affiliations:** 1 Department of Computer Science and Technology, School of Computer Science, Northeast Electric Power University, Jilin 132013, China; smile2867ling@neepu.edu.cn (L.W.); 2020307030101@neepu.edu.cn (C.D.); 2202000719@neepu.edu.cn (Y.Z.); 2 Key Laboratory of Computing Power Network and Information Security, Ministry of Education, Shandong Computer Science Center (National Supercomputer Center in Jinan), Qilu University of Technology (Shandong Academy of Sciences), Jinan 250000, China; dingw@sdas.org; 3 Shandong Provincial Key Laboratory of Computer Networks, Shandong Fundamental Research Center for Computer Science, Jinan 250000, China; 4 Data Science Laboratory, Faculty of Information Technology, Ton Duc Thang University, Ho Chi Minh City 700000, Vietnam; khryu@chungbuk.ac.kr; 5 Research Institute, Bigsun System Co., Ltd., Seoul 06266, Republic of Korea; 6 Database and Bioinformatics Laboratory, College of Electrical and Computer Engineering, Chungbuk National University, Cheongju 28644, Republic of Korea; 7 Department of Computer and Information Engineering, Kunsan National University, Gunsan 54150, Republic of Korea; kwnam@kunsan.ac.kr

**Keywords:** video behavior recognition, lightweight skeleton feature extraction, trajectory prediction, occlusion action recognition, data mining

## Abstract

Video action recognition based on skeleton nodes is a highlighted issue in the computer vision field. In real application scenarios, the large number of skeleton nodes and behavior occlusion problems between individuals seriously affect recognition speed and accuracy. Therefore, we proposed a lightweight multi-stream feature cross-fusion (L-MSFCF) model to recognize abnormal behaviors such as fighting, vicious kicking, climbing over the wall, et al., which could obviously improve recognition speed based on lightweight skeleton node calculation, and improve recognition accuracy based on occluded skeleton node prediction analysis in order to effectively solve the behavior occlusion problem. The experiments show that our proposed All-MSFCF model has a video action recognition average accuracy rate of 92.7% for eight kinds of abnormal behavior recognition. Although our proposed lightweight L-MSFCF model has an 87.3% average accuracy rate, its average recognition speed is 62.7% higher than the full-skeleton recognition model, which is more suitable for solving real-time tracing problems. Moreover, our proposed Trajectory Prediction Tracking (TPT) model could real-time predict the moving positions based on the dynamically selected core skeleton node calculation, especially for the short-term prediction within 15 frames and 30 frames that have lower average loss errors.

## 1. Introduction

Video surveillance technology is widely used in daily life for public safety management. Abnormal behavior recognition and tracking technology, as an important video surveillance application technology, has a great deal of research significance and practical value. Abnormal behavior has different definitions in different scenarios, and in our research the abnormal behavior studied is based on the field of public security. According to the definition of “behavior” and “abnormal”, the abnormal behavior classification is clarified. “Behavior” [1] refers to the most basic and meaningful interactions with their surrounding environment. “Abnormal” [2] refers to phenomena that are different from the normal state. Therefore, the following definition of “abnormal behavior” is given: all actions, gestures, or events made in the current scene that are not suitable.

Abnormal behavior recognition and tracking technology can improve the efficiency and accuracy of video surveillance, reduce operators’ workloads, and detect and handle abnormal behavior events early. Abnormal recognition and tracking based on skeleton nodes is one of the important methods in current research. The detection effect of skeleton nodes is continuously improved, and it is increasingly becoming one of the core technologies of intelligent video surveillance. However, in real application scenarios, the large number of skeleton nodes or the occlusion between individuals seriously affects the abnormal behavior recognition speed and accuracy, and limits the application of abnormal behavior recognition and tracking algorithms. Therefore, we propose the LSFE model. Before feature extraction, for the problem of occluded skeleton nodes information, the *L*-MSFCF model is proposed to utilize the skeleton nodes information of past frames to predict the occluded skeleton node information, thus improving accuracy. When tracking abnormal targets, in order to address the issue of targets becoming occluded or disappearing from view, we suggest the TPT model.

The structure of this paper is as follows: The history of the current quest for abnormal behavior recognition is provided in Section 1, and the current status of the field is introduced in Section 2. The study’s purpose, materials, and procedures are all presented in Section 3. The LSFE approach is described in full in Section 4. A comprehensive explanation of the *L*-MSFCF model is given in Section 5. The TPT model is explained in depth in Section 6. The findings and analysis of the experiment are presented in Section 7. A thorough discussion is given in Section 8, and the study is concluded in Section 9.

## 2. Related Work

Computer vision techniques are gradually becoming the mainstream of abnormal behavior recognition, and the main challenge is to accurately extract and analyze representative appearance features and dynamic motions. In the early stages of the study, researchers typically thought of objects as particles. By simulating the tension in every pixel, Mehran R et al. [3] created a particle flow network to extract the interaction of force as features from video data. In order to capture the space-time properties of a crowd, new global features were proposed by Xie S et al. [4] to describe the position, speed, and direction of particles. Furthermore, Yu B et al. [5] enhanced the representation capacity of the particles by utilizing several comparable particles to describe objects. However, these feature-extraction techniques were unable to recover the subtle aspects of motions. In order to better capture motion information, numerous researchers have turned to feature extraction from space-time cubes, whereas Sabokrou M et al. [6] considered the sub-region of continuous frames as a space-time cube and extracted 3D gradient characteristics for cubes, and Fayyaz M et al. [7] collected global features from space-time cubes using an automatic encoder. Martinel N et al. [8] extracted deep features by rebuilding the interesting cubes using stacked sparse automatic encoders. Since features extracted from space-temporal cubes do not maintain the correlation of motion features between the cubes, Coşar S et al. [9] learned velocity and trajectory from real tracking data pixels and clustered the trajectories using a clustering tree to predict the most probable paths of the tracked objects. Xu M et al. [10] captured both groups and personal trajectories at the same time, and performed separate abnormal behavior detection.

Target motion trajectory prediction algorithms are vital to research in computer vision and robotics areas. The purpose is to forecast a target’s future motion trajectory utilizing existing motion data and environmental information so that a robot or other intelligent system can react appropriately. Kerdvibulvech C et al. [11,12] proposed a method for 3D human motion analysis for reconstruction and recognition. They used 3D gait signatures computed from 3D data that are obtained from a triangulation-based projector–camera system. Results demonstrated that the proposed 3D gait signatures-based biometrics provide valid results on real-world 3D data. Combining trajectory prediction due to maneuver recognition with trajectory prediction owing to constant slew rate and acceleration motion models was conducted by Houenou A et al. [13] and Czyz J et al. [14] proposed a mixed-value sequence state estimation algorithm. Shao X et al. [15] presented a unique filtering technique, which is to follow a target’s mobility utilizing GPS sensors. Vashishtha D et al. [16] and Kapania S et al. [17] improved particle filtering, combined color sequences, and constrained Bayesian state estimation to achieve motion trajectory prediction of the target. Choi D et al. [18] proposed a method using maximum likelihood multi-filter to obtain an overall estimate to predict the target trajectory by combining independent multiple kinematic model correlation estimates through a great likelihood rule. Predicting target trajectories by building kinematic and kinetic models will not affect the accuracy even if losing a large part of the data, but part of the target motion trajectory is nonlinear and prone to many curvilinear trajectories, which means that the model-based trajectory prediction algorithms will have the problem of low accuracy. Another important model is based on data-driven trajectory prediction studies [19]. The model uses both classification and regression algorithms to treat the trajectory prediction issues. Semwal et al. [20] suggested a target trajectory prediction technique, long short-term memory networks (LSTMs), and convolutional neural networks (CNNs). Deep neural network by Shirazi M S et al. [21], Faster R-CNN by Zhou H et al. [22], and YOLO network by Yoon Y C et al. [23] also have good performance in target trajectory prediction.

To solve the occlusion problem, Sabokrou M et al. [24] first applied fully convolutional neural networks to abnormal behavior detection. They completed it by utilizing AlexNet’s fully convolutional layer to extract deep features and by cascading Gaussian classifiers to identify abnormal behaviors. Chu W et al. [25] extracted temporal characteristics using 3D convolutional neural networks. Liqian Yan [26] suggested a 3D convolutional residual network structure in light of this. In order to lessen the effect of the network, Fang Z et al. [27] defined the motion characteristics of the footage using a visual system to define spatial features and the multi-scale histogram of the optical flow. Ye O et al. [28] extracted initial features through the CNN-LSTM network and used feature expectation subgraph to filter unexpected feature values. The values of the remaining predicted features were sent into SVM to detect abnormal behavior, and Tay N C et al. [29] created a shallow convolutional neural network to extract appearance characteristics, added spatial attention, and integrated it with a LSTM network.

In previous conclusions, although the target trajectory prediction and occlusion problems can be solved effectively, the accuracy and time complexity need to be improved. Therefore, we need further research on abnormal behavior recognition.

## 3. Materials and Methods

### 3.1. Motivation

**Figure 1:**
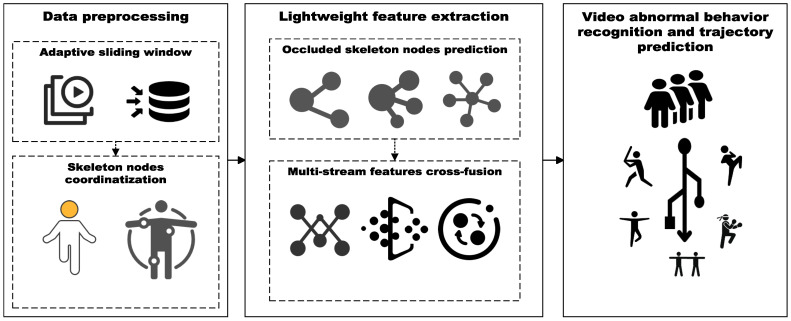
Abnormal behavior recognition and tracking flowchart.

In our research, lightweighting the skeleton is an effective way to cope with the effects of an excessive number of skeleton nodes. In addition, we found that the multi-stream feature cross-fusion method has significant advantages in feature extraction. Therefore, the flowchart of abnormal behavior recognition and tracking is demonstrated in Figure 1.

### 3.2. Datasets

The experiment utilized the human3.6m dataset, comprising 3.6 million 3D human posture examples and their related photos. These data were collected from six males and five females across 17 diverse scenes such as discussions, smoking, taking photos, and more. The video captures were from four calibrated cameras capable of capturing precise 3D joint positions and joint angles. For more information about the human 3.6 m dataset, see [30]. The UCF-Crime dataset [31], a vast collection of actual surveillance footage containing 1900 long, unedited recordings with 13 distinct kinds of abnormal events, was also used in the studies. Furthermore, the ShanghaiTech Campus dataset [32] was employed. It included over 270,000 training frames and 130 occurrences of abnormal events.

### 3.3. Methods

This paper is a study of abnormal behavior recognition and tracking in surveillance videos. First, the skeleton nodes of various behaviors are lightened by the LSFE method and then construct the optimal skeleton node architecture graphs for various behaviors. Second, they construct the *L*-MSFCF model for abnormal behavior recognition; after predicting the information of the occluded skeleton nodes, it takes the lightweight feature skeleton to coordinate information and the skeleton vectors as the dual-stream inputs and uses the cross-feature fusion to carry out the feature extraction. Finally, the TPT model is proposed for trajectory prediction. It provides a reference for the tracking of abnormal behavior targets.

## 4. Skeleton Feature Extraction

We proposed a lightweight skeleton feature extraction (LSFE) method to solve the problem of a large number of skeleton nodes. Firstly, we design the adaptive computation of the video frame window and find out the optimal video frame window length for optimizing the skeleton nodes; secondly, we design the formula for calculating the skeleton nodes of the proposed trigonometry and triangulated all the skeleton nodes, and find out the motion law of the individual behavioral movement process by using the association rule mining algorithm under the optimal video frame window length; finally, we find out the skeleton nodes that can represent the action by data mining and filter out the redundant skeleton nodes, so as to achieve the purpose of skeleton nodes optimization.

### 4.1. Data Preprocessing

The definition of behavior in this paper divides behavior into two categories: normal behavior and abnormal behavior. We constructs a normal behavior video database and an abnormal behavior video database. The definitions are as follows (Table 1).

**Figure 2:**
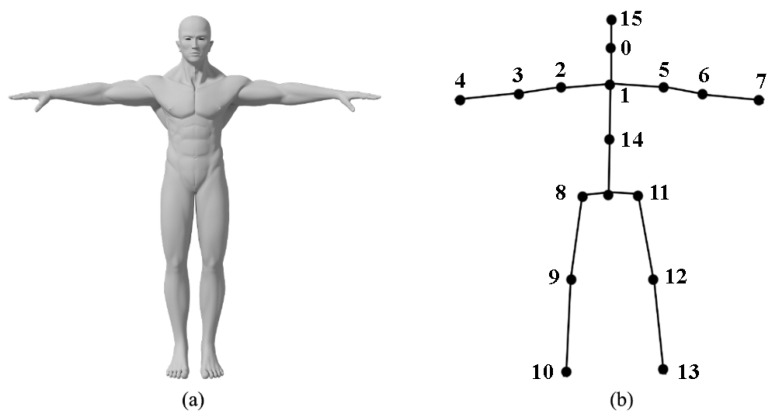
Skeleton node transformation schematic diagram. (**a**) Video data diagram; (**b**) skeleton data diagram.

Before action recognition based on skeleton joint point, it is necessary to convert the original video data into skeleton joint data, the space representation of the action is detected and recognized in the original video data. Through the existing posture evaluation algorithm, the video data can be transformed into corresponding skeleton joint data and the skeleton corresponding to each number in Figure 2 is represented as follows: 0—nose, 1—neck, 2-right shoulder, 3—right elbow, 4—right hand, 5—left shoulder, 6—left elbow, 7—left hand, 8—right hip, 9—right knee, 10—right foot, 11—left hip, 12—left knee, 13—left foot, 14—stomach, and 15—head.

**Figure 3:**
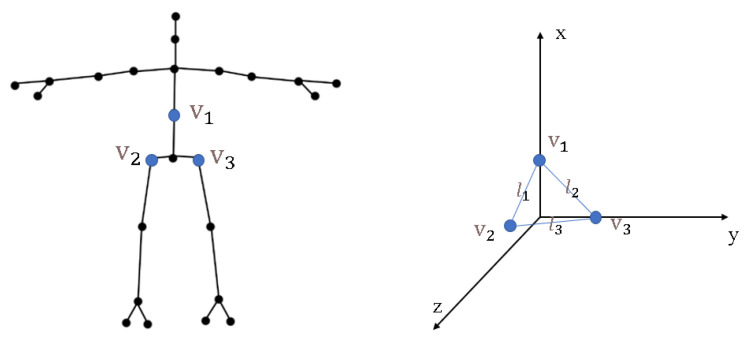
Core skeleton node triangle calculation in 3D coordinates.

Due to the different angular positions of the sportsman relative to the camera, resulting in possible differences in the coordinate origin, to facilitate the study, we do a harmonized coordinate transformation for the skeleton data. We reconstruct the coordinate with a triangle formed by the three points $v_{1}$, $v_{2}$, and $v_{3}$ in Figure 3.

$v_{1}$, $v_{2}$, $v_{3}$ in space constitute a triangle and its three sides are $l_{1}$, $l_{2}$, $l_{3}$, $v_{t}$, set as the projection point on the line $l_{3}$. Through Equation (1) we can obtain three basis vectors by transforming the coordinate.

(1) $$\begin{array}{c} U_{t}=\left(\frac{\left(v_{3}-v_{t}\right)⊗\left(v_{1}-v_{t}\right)}{\|v_{3}-v_{t}\left\|⊙\right\|v_{1}-v_{t}\|},\frac{v_{3}-v_{t}}{\|v_{3}-v_{t}\|},\frac{v_{1}-v_{t}}{\|v_{1}-v_{t}\|}\right) \end{array}$$

$v_{1},v_{2},v_{3}$—the three skeleton joints in Figure 3;

$v_{t}$—the projection point on the line $l_{3}$;

$U_{t}$—the three basis vectors of the transformed coordinate.

The conversion process of the coordinates also needs the three basis vectors of the original coordinate. It is represented in Equation (2).

(2) $$\begin{array}{c} U_{0}=\left[\begin{array}{ccc} 1 & 0 & 0\\ 0 & 1 & 0\\ 0 & 0 & 1 \end{array}\right] \end{array}$$

Using Equation (3), the three basis vectors of the original coordinate and the three basis vectors of the transformed coordinate are operated to obtain the corresponding transformation matrix.

(3) $$\begin{array}{c} R=U_{t}^{-1}⊗U_{0} \end{array}$$

$U_{t}^{-1}$—the inverse matrix of $U_{t}$.

Using the transformation matrix, the original coordinates are transformed by Equation (4).

(4) $$\begin{array}{c} \left(\begin{array}{c} v\\ 1 \end{array}\right) \end{array}=\left(\begin{array}{cc} R & v_{1}\\ 0 & 1 \end{array}\right)\left(\begin{array}{c} v^{\prime }\\ 1 \end{array}\right)$$

*v*—a skeleton node in the original coordinate;

$v^{\prime }$—the corresponding transformation node;

*R*—transformation matrix;

$v_{1}$—new coordinate origin.

During the transformation, we designate the origin of the new coordinate as $v_{1}$ in the existing coordinate; moreover, all 3D skeleton coordinates are transformed into the new skeleton data with $v_{1}$ as the origin by the above equation.

### 4.2. Adaptive Sliding Window Selection Calculation

**Figure 4:**
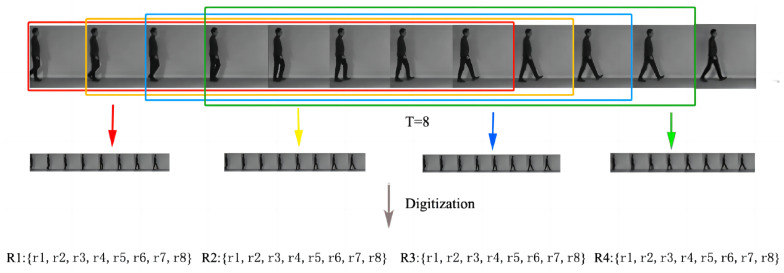
Adaptive sliding window intervals selection calculation example.

Activities are characterized by continuity and periodicity. However, the length of this cycle cannot be determined; therefore, this paper utilizes adaptive sliding window selection calculation to determine the cycle length that meets the requirements. First, we apply the method to the segmentation of action sequences. For the action sequence $a=\left(\begin{array}{cccc} a_{1}, & a_{2}, & \cdots & a_{n} \end{array}\right)$, we set the window width to *T* and step size to *K*, and each window contains $R_{i}=\left(\begin{array}{cccc} r_{i1}, & r_{i2} & \cdots & r_{iT} \end{array}\right)$. In this way, the initial action sequence is divided into FT action segments, which can be represented as $R=\left(\begin{array}{cccc} A_{1}, & A_{2} & \cdots & A_{FT} \end{array}\right)$. Each segment contains *K* poses describing the local information of the body. Figure 4 shows the complete process of segmenting an action sequence by adaptive sliding window selection calculation and the original video frames are cited by the Human3.6m dataset [30].

The window width parameter *T* determines the size and the number of segments that can be segmented in an action sequence. A larger *K* means that each segment contains more poses and a coarser description of the movement; on the contrary, a smaller *K* means that each segment contains fewer poses and a more accurate description of the movement. Although smaller *K* describes movements more accurately, this means that smaller segments are more susceptible to noise in the 3D skeleton position tracking results, which in turn affects the recognition of movements. Defining the set of stored window sizes as *L*, we calculate each action accuracy in the window size from 3 to 23. The top three action accuracies are stored in the set *L*; we can obtain the calculation of all the actions and then take the size of the window with the highest number of occurrences as the window length. Finally, the most frequent occurrence is 15. Therefore, the window length of T=15 is taken as a basic action sequence in this paper.

### 4.3. Lightweight Skeleton Feature Extraction Method (LSFE Method)

We put forward the lightweight skeleton feature extraction (LSFE) method. The method is based on the association rule mining of similar vectors. It converts the 3D skeleton data into a series of vectors with a length of 15 frames by using adaptive sliding window selection calculation and then utilizes vector similarity to mine the similarity association rule set of each node. If there exists a similarity association rule set, the skeleton node is considered to be a strongly associated skeleton node of the action. The computation is as follows:

Step 1: Obtain any one of its skeleton nodes $v_{i}$ based on the original skeleton node data *V*. Define the node data of the last two frames as $v_{i+1}$, $v_{i+2}$. Define the 3D coordinates of $v_{i}$, $v_{i+1}$, $v_{i+2}$ as $\left\{\begin{array}{ccc} x_{i} & y_{i} & z_{i} \end{array}\right\},\left\{\begin{array}{ccc} x_{i+1} & y_{i+1} & z_{i+1} \end{array}\right\},\left\{\begin{array}{ccc} x_{i+2} & y_{i+2} & z_{i+2} \end{array}\right\}.$

Step 2: Calculate the angle change in the skeleton nodes $v_{i}$, $v_{i+1}$, $v_{i+2}$ and the three points in the time dimension according to Equation (5).

(5) $$\left\{\begin{array}{c} \alpha =\tan^{-1}\left(\frac{|x_{i}-x_{i+2}|}{|z_{i}-z_{i+2}|}\right)\\ \beta =\tan^{-1}\left(\frac{|x_{i+1}-x_{i+2}|}{|z_{i+1}-z_{i+2}|}\right)\\ \theta =\tan^{-1}\left(\frac{|x_{i}-x_{i+1}|}{|y_{i}-y_{i+1}|}\right) \end{array}\right.$$

α, β, θ—the value of the angle change;

The plane ρ can be obtained through the skeleton nodes $v_{i}$, $v_{i+1}$, $v_{i+2}$, which are represented as shown in Equation (6).

(6) ρ:ax+by+cz+d=0

*a*, *b*, *c*, *d*—the plane equation parameters;

It is clear that the normal vector $n=\left(\begin{array}{ccc} a, & b, & c \end{array}\right)$ of the plane ρ can be obtained, then the distance *R* from the origin of the space coordinates to this plane is obtained through Equation (7).

(7) $$R=\frac{|d|}{\sqrt{a^{2}+b^{2}+c^{2}}}$$

After obtaining the height *R* of the proposed triangular pyramid, the volume $X_{i}$ is then calculated according to Equation (8).

(8) $$X_{i}=\frac{1}{3}\times 2\times R\times \sin\frac{\alpha }{2}\times \sin\frac{\beta }{2}\times \sin\frac{\theta }{2}$$

α, β, θ—the three included angles of the proposed triangle pyramid;

*R*—height of the proposed triangular pyramid;

$X_{i}$—volume of the proposed triangular pyramid.

According to Equation (8), we can find the set of vectorized data $X_{j,M_{i}}=\left\{X_{i}\right\}$ for a certain skeleton node $v_{i}$ of a certain action single video $M_{j}$, then the vectorized dataset for all the data of a certain skeleton node of the action is denoted as Equation (9).

(9) $$M_{i}=\left\{\left\{X_{1,M_{1}}\right\},\left\{X_{2,M_{2}}\right\}\left\{X_{j-1,M_{i-1}}\right\},\left\{X_{j,M_{i}}\right\}\right\}$$

$X_{j,M_{i}}$—the vectorized dataset of the *i*th skeleton node of the *j*th video data under the action classification;

$M_{i}$—a vectorized set of all data for a certain skeleton node.

Step 3: Construct the frequent item set. Scan all the $\left\{X_{i}\right\}$ data in the $M_{i}$ set in a single pass to determine the support of each $\left\{X_{i}\right\}$. Since $\left\{X_{i}\right\}$ is a vector datum, it utilizes a calculation rule of similar vectors: if two vectors are similar vectors then their frequency adds one. The similar vectors are shows in Equation (10).

(10) $$\cos\delta =\frac{\Sigma_{t=1}^{n}\left(\left\{X_{a}\right\}*\left\{X_{b}\right\}\right)}{\sqrt{\Sigma_{t=1}^{n}X_{a}^{2}*\sqrt{\sum_{t=1}^{n}X_{b}^{2}}}}$$

$X_{a}$—data *a* of a certain skeleton node in the frequent item set;

$X_{b}$—data *b* of a skeleton node in the frequent item set;

cosδ—similarity of vectors $X_{a}$ and vectors $X_{b}$.

$X_{a}$ and $X_{b}$ are two vectors of the same length, cosδ is between 0 and 1. When cosδ > 0.9, the vector $X_{a}$ is considered to be similar to the vector $X_{b}$.

Step 4: Mining association rule sets. Define the association rule set of an individual’s behavior as a set in the form of key-value pairs $J=\{\left(v_{i}:Y_{i}\right)|v_{i}\in V,Y_{i}\neq \emptyset \}$. Mine the 16 skeleton nodes of an individual to obtain the set of association rules *L* for a single node $v_{i}$, and *L* is added to $Y_{i}$. When $\;Y_{i}=\emptyset \;,\;\left(v_{i}:Y_{i}\right)\;$, $\left(v_{i};Y_{i}\right)$ will be stored in the association rule set *J* as key-value pairs; $Y_{i}=\emptyset$ means that the current skeleton nodes do not have an obvious regularity; they cannot represent the behavior action and should be discarded. Define the final association rule set $J^{\prime }=\left\{\left(v_{i}:Y_{i}\right)|v_{i}\in V,Y_{i}\neq \emptyset \right\}$, the set of all skeleton nodes in the set $J^{\prime }$ is $\left\{v_{i}\right\}$, $\left\{v_{i}\right\}$ is a non-empty subset of *V*.

Step 5: Determine lightweight skeleton nodes. The maximum frequent item set in $J^{\prime }$ is *n*. When n>μ, then $v_{i}$ is considered as the skeleton node of the current action. Finally, calculate all feature skeleton nodes.

The highest accuracy of skeleton node recognition is when μ=3. The extracted lightweight skeleton nodes for each action are shown in Table 2.

Step 6: Based on the above lightweight skeleton nodes, construct the LSFE model to recognize actions and verify the feasibility of lightweight skeleton nodes.

## 5. Lightweight Multi-Stream Features Cross-Fusion Model (*L*-*MSFCF* Model)

Lightweight feature skeleton node extraction is a core processing step for supporting the *L*-MSFCF model, which could help to greatly reduce the model parameter numbers and computation time than full skeleton processing. In fact, as shown in the experiment Section 7.2 (Section 4—LSFE model testing), only considering the optimized skeleton nodes to recognize the video behaviors, the accuracy is not ideal. In order to improve the recognition accuracy and further reduce the computation time, our proposed *L*-MSFCF model has enhanced lightweight features based on a multi-stream feature cross-fusion process in order to obtain more behavior feature information.

### 5.1. L-MSFCF Model Abnormal Behavior Recognition Process

The *L*-MSFCF model is different from the traditional multi-stream feature fusion action recognition method. The *L*-MSFCF model processes the occluded skeleton nodes and also utilizes the feature cross-fusion extraction method. Firstly, lightweight the skeleton nodes. Secondly, predict the occluded skeleton node information by utilizing the skeleton node data information of past frames. Finally, obtain action features through the skeleton stream, nodes stream, and feature cross-fusion stream. The *L*-MSFCF model strengthens the recognition ability of abnormal behaviors.

**Figure 5:**
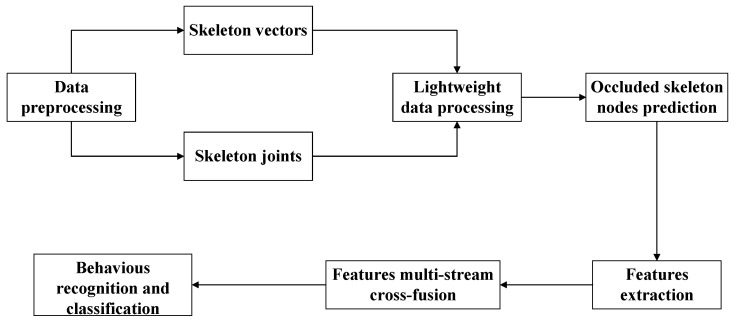
*L*-MSFCF model flowchart.

The *L*-MSFCF model abnormal behavior recognition process mainly has two steps: the first step is occluded skeleton nodes prediction and lightweight processing; the second part is lightweight skeleton data feature extraction through dual-stream, and then feature fusion is performed on all the features to finally obtain the classification results. Figure 5 shows the flowchart of the *L*-MSFCF model. The following are the steps:

Step 1: Preprocessing the skeleton data. Create a skeleton joint dataset and a skeleton vector dataset. Because skeleton vectors are composed of two skeleton nodes and the whole skeleton point graph is not a ring structure, this results in the number of skeleton vectors always being less than the number of skeleton nodes in the generation process by 1. We add an empty skeleton with the value of 0 to skeleton vectors so that there are as many skeleton nodes.

Step 2: Lightweighting the skeletons. Lightweight skeleton data are based on lightweight characteristic skeleton nodes for each action. Skeleton node data and skeleton vector data are processed similarly, taking skeleton node data processing as an example. The process is as follows:

According to Table 2, retain the corresponding characteristic skeleton node information and set other skeleton information to 0. Take fighting as an example; its original skeleton data of a certain frame is expressed as Equation (11).

(11) $$\begin{array}{c} v_{ti}=\left[g_{1},g_{2},g_{3},g_{4},g_{5},g_{6},g_{7},g_{8},g_{9},g_{10},g_{11},g_{12},g_{13}\right] \end{array}$$

$V_{ti}^{\prime }$—the original skeleton dataset of a frame;

*g*—a skeleton node in the current frame.

The lightweight skeleton nodes for the fighting in Table 2 are [3, 4, 6, 7, 9, 10, 12, 13], and the result of lightweight processing is shown in Equation (12).

(12) $$\begin{array}{c} v_{ti}^{\prime }=\left[0,0,g_{3},g_{4},0,g_{6},g_{7},0,g_{9},g_{10},0,g_{12},g_{13}\right] \end{array}$$

$v_{ti}^{\prime }$—skeleton dataset after lightweight processing;

*g*—a skeleton node in the current frame.

Step 3: Determine whether the lightweight skeleton node data are occluded or not; if the space coordinates of this skeleton node data are all 0, it is determined that this skeleton node data are occluded, then the skeleton node data are predicted.

Step 4: Process the skeleton node data and skeleton vector data separately by convolution to obtain features that can represent each action.

Step 5: Combine the skeleton node features and skeleton vector features to form the overall action features utilizing feature fusion.

### 5.2. Occluded Skeleton Node Prediction

Occluded skeleton nodes can cause noise to the abnormal behavior recognition, affecting the accuracy. To solve the problem, we suggest a generative network-based method for occluded skeleton node prediction, which utilizes the skeleton node data from past frames to predict the skeleton node information of the next frame.

The advantages over existing methods are: the GRU at the lowest level can learn the motion information of the smallest unit frame without interference from higher levels, and the higher levels can capture different features of the motion of specific length frames; moreover, the latest GRU outputs from different levels are used as inputs during the prediction period of each time step, which makes the motion information more adequate and the features of the next frame more comprehensive.

**Figure 6:**
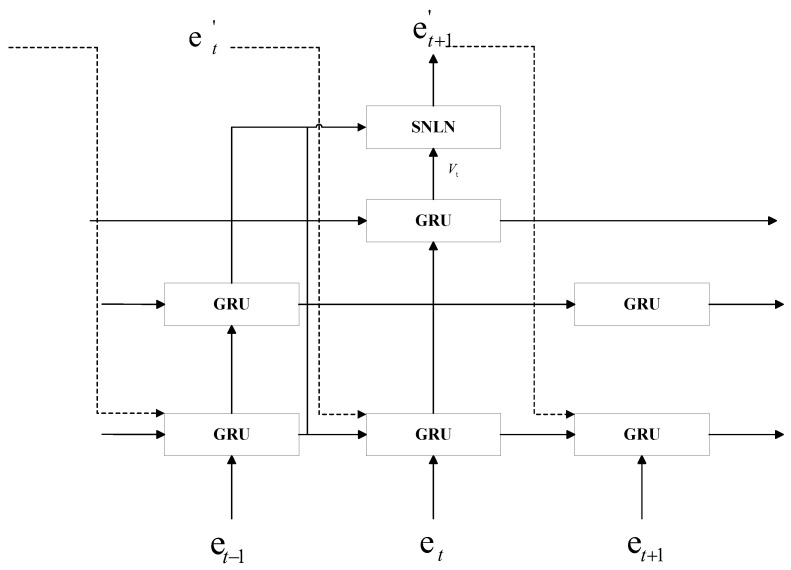
Occluded skeleton node prediction architecture.

In Figure 6, the skeleton data information of the previous, the current, and the future frame is represented by the vectors $e_{t+1},e_{t},e_{t+1}$. The expected skeleton data information at moments *t* and t+1 is $e_{t}^{\prime }$ and $e_{t+1}^{\prime }$. The skeleton data information for every time step is used as a series of input GRU units at the first layer. Define *K* distinct GRU unit sequences at the second level, each of which will only accept similar inputs from the first level’s GRU units that have been time-step-modeled. If K=2, for instance, the second layer would contain two GRU sequences: the first would be derived from time frame data t={1,3,5,⋯}, whereas the second would come from time frame data t={2,4,6,⋯}. GRUs at the same hierarchical level share weights, improving the characteristics of the skeleton data to improve long-term dependent learning. There are a total of $K^{2}$GRU sequences on the third layer because for every *K*GRU sequence on the second layer, there are *K* different GRU sequences corresponding to it in the third layer. Each GRU sequence uses the same complex modulo *K* inputs from it. Up to level (M−1), where a GRU sequence of $K^{M-1}$ will exist in level *M*, the hierarchy’s process of creating new, higher-level GRU sequences continues. In order to produce skeleton vector predictions for the associated hidden units in all hierarchies, a two-layer connected network is finally introduced. The inputs for these projected skeleton vectors will then contribute to the skeleton vector prediction process for upcoming frames.

### 5.3. Lightweight Multi-Stream Feature Cross-Fusion Process

Behavior recognition method networks with multi-stream feature fusion, such as dual-stream networks, 2s-AGCN [33], typically utilize single-stream networks to extract characteristics independently before fusing them. The feature fusion method performs weight fusion at the end, and the average pooling layer will overrun the fusion step, making the network unable to fully perform each dependent feature.To solve this question, this subsection proposes a *L*-MSFCF model, which performs feature cross-fusion during pooling to fully utilize each tributary feature. There are two parts to introduce the model: the network architecture and the basic convolution module.

The whole network of *L*-MSFCF consists of three sub-stream networks: skeleton vector stream network, skeleton joint stream network, and features cross-fusion stream network. Each sub-stream network utilizes the 2s-AGCN graph convolution network as the backbone. Either joints or skeletons can be used as input data. Formally, the skeleton sequence data are $V=R^{C\times T\times S}$, DϵC×T×S, and *C*, *T*, *S* separately denote channel dimension, time dimension, and space dimension. Space characteristics may be extracted from the input data via the spatial stream network. Shallow sub-networks have a lot of inaccurate and localized data in their features. Conversely, features located in the network’s deeper levels have less false information and more global information. Many conventional networks are bottom-up and end-to-end systems that only employ a subset of top-layer characteristics. These methods lack local information that facilitates action recognition classification. For this reason, the network proposed in this paper selects features from multiple layers. The features extracted from different levels have different feelings and contain various local and global information.

The whole process of feature fusion is as follows:

Step 1: Mark the skeleton vector features collected from the skeleton vector stream network, denoted as $f_{bv}^{1},f_{bv}^{2},f_{bv}^{3},\cdots ,f_{bv}^{L}$. The skeleton joint stream network is almost identical to the skeleton stream network, where the extracted features are, respectively, denoted as $f_{bn}^{1},f_{bn}^{2},f_{bn}^{3},\cdots ,f_{bn}^{L}$. *L* is the maximum layer of elements. In the experiment part, we set *L* to 3.

Step 2: Calculate the weights of the skeleton vector stream network and the skeleton joint stream network. The skeleton vector stream network $N_{bv}\left(D\right)$ and the skeleton joint stream network $N_{bn}\left(D\right)$ are represented as shown in Equations (13) and (14).

(13) $$\begin{array}{c} N_{bv}\left(D\right)=\left(f_{bv}^{1},f_{bv}^{2},\cdots ,f_{bv}^{L}\right)\cdot p_{bv} \end{array}$$

(14) $$\begin{array}{c} N_{bn}\left(D\right)=\left(f_{bn}^{1},f_{bn}^{2},\cdots ,f_{bn}^{L}\right)\cdot p_{bn} \end{array}$$

$N_{bv}\left(D\right)$—skeleton vector stream network;

$N_{bn}\left(D\right)$—skeleton joint stream network;

$P_{bv}$—skeleton vector stream network weight;

$P_{bn}$—skeleton joint stream network weight.

Step 3: The fusion stream network inputs the features collected from the basic dual-stream network, and the weights of the fusion stream network are calculated. As an example, for the case where *L* is 3, the fusion stream network is represented as shown in Equation (15).

(15) $$\begin{array}{c} N_{fus}\left(f_{bv}^{1},f_{bv}^{2},f_{bv}^{3},f_{bn}^{1},f_{bn}^{2},f_{bn}^{3}\right)=p_{fus} \end{array}$$

$N_{fus}$—fusion stream network;

$p_{fus}$—fusion stream network weight.

Step 4: Use weighted average fusion function w(·) to compute the prediction weight of the whole network.

(16) $$\begin{array}{c} w\left(p_{bv},p_{bn},p_{fus}\right)=\frac{\alpha p_{bv}+\beta p_{bn}+\gamma p_{fus}}{\alpha +\beta +\gamma } \end{array}$$

α,β,γ—weighted average fusion function fixed weight parameters.

Step 5: The feature data of the three tributaries are fused in the fusion layer by weighted average fusion, and finally in the fully connected layer by Softmax function. Fuse all the information to finally output a feature that can represent the whole action.

**Figure 7:**
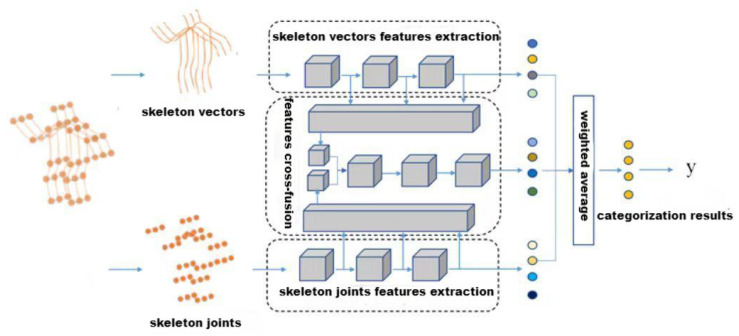
*L*-MSFCF model architecture.

The convolution module’s goal is to extract deep features. This paper utilizes an adaptive graph convolutional network, and the advantage is that the whole process is a bottleneck structure, aiding in first reducing noise and then obtaining extremely effective information. Its specific structure is shown in Figure 7.

The entire convolutional block can be represented as:

(17) $$\begin{array}{c} f_{out}=\sum_{k}^{K_{v}}W_{k}f_{in}\left(A_{k}+\delta C_{k}\right) \end{array}$$

$f_{in}$—input features;

$f_{out}$—output features;

$K_{v}$—kernel size in space dimensions;

$W_{k}$—1 × 1 convolution operation;

$A_{k}$—N × N adjacency matrix, its elements indicate whether a vertex is in a subset of another vertex;

δ—weighting parameter;

$B_{k}$—data-driven matrix.

**Figure 8:**
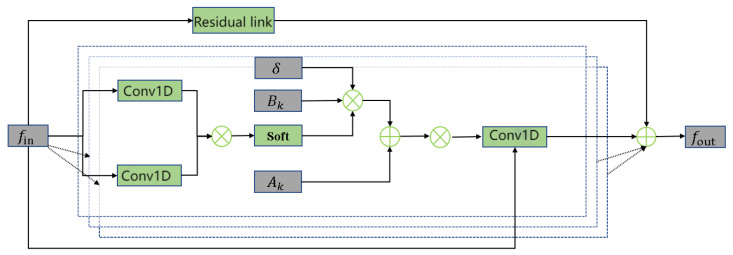
*L*-MSFCF convolutional block.

Throughout the computation, we set the $K_{v}$-space dimension’s kernel size to 3. $A_{k}=\rho {\hat{A}}_{k}\rho$, and $A_{k}$ is N × N adjacency matrix whose elements indicate whether the weak feature skeleton nodes are in the subset of lightweight feature skeleton nodes or not. ρ is the normalized diagonal matrix, $\rho_{k}=\Sigma_{j}{\hat{A}}_{k}+\sigma$. δ is set to 0.001 to avoid blank lines. $W_{k}$ denotes a 1 × 1 convolution operation with weights in the shape of $B_{k}\times B_{k}\times 1\times 1$. $B_{k}$ is a data-driven matrix in the shape of N × N. $B_{k}\times B_{k}$ is a non-local block that goes through the computation of Figure 8 once before participating in a second computation. The value of δ directly determines the impact of $B_{k}$ on the quadratic convolution. In the experiment, we set δ = 0.3 to obtain high-level valid information, and if the parameters and elements in the matrix were not initialized, its value was set to 0.01.

## 6. Trajectory Prediction Tracking Model (TPT Model)

### 6.1. Five-Bit Skeleton Screening Method

**Figure 9:**
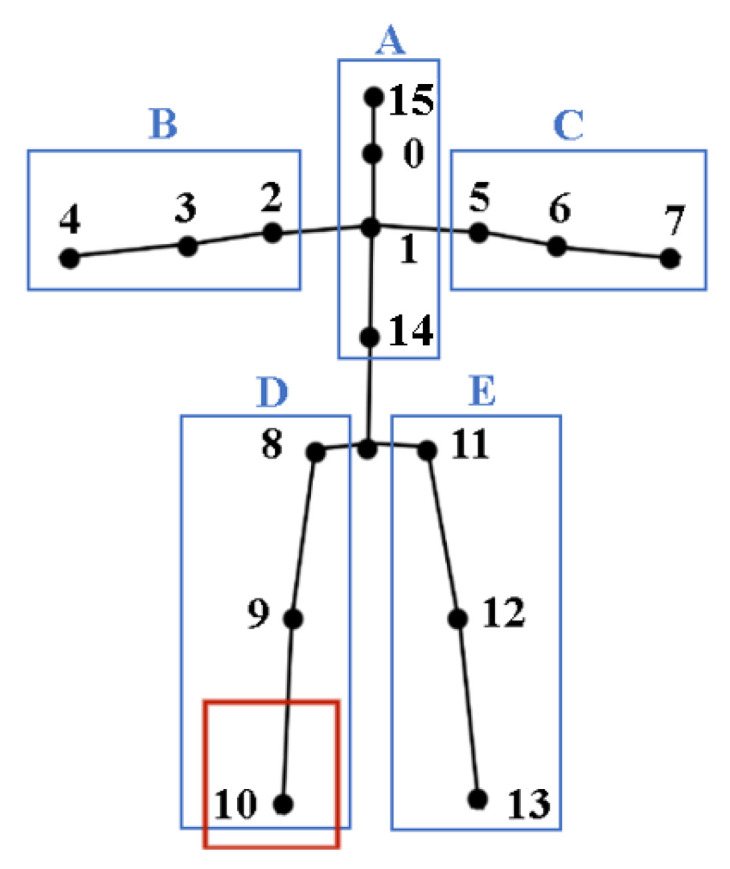
Skeleton node partitions.

To lower the entire model’s time complexity and reduce the impact of skeleton node occlusion on trajectory prediction, this paper proposes a five-bit screening method. First, the skeleton nodes are divided into five parts, A, B, C, D, and E, and their partitions are in Figure 9. Then, the skeleton nodes in each partition are sorted, utilizing lightweight feature skeleton node extraction results in Table 2. After that, select the feature skeleton nodes that can represent each partition. Finally, find out the mass point that is based on the five featured skeleton data, regard it as the starting point for trajectory prediction tracking.

Calculate the probability of their occurrence based on feature skeleton node extraction results in Table 2, and sort the skeleton nodes in each partition by top; the results are as follows:

We select the characteristic points in each partition and then denote them as $e_{A}$, $e_{B}$, $e_{C}$, $e_{D}$, $e_{E}$. Form a pentagon $e_{x}$ by the five points. The calculation is in Equation (18).

(18) $$\begin{array}{c} v_{x}=\frac{1}{N_{v}}\Sigma_{1}^{N_{v}}v_{i} \end{array}$$

$N_{v}$—vertices number;

$v_{i}$—vertices space coordinates;

$v_{x}$—mass point space coordinates.

In most cases, we can essentially detect complete skeleton nodes. However, in some cases, some are not detected. For example, the skeleton node 10 is occluded in the partition D of Figure 9. To solve this problem, we can select the top-ordered skeleton nodes of the partition in turn, and according to Table 3, node 9 should be selected as the representative node of the partition. When a partition is occluded, set it to the same position detected in the previous frame.

Considering that trajectory is a vector with velocity and direction, this paper calculates the change of direction and velocity of the mass point $e_{x}$ for each frame. The velocity of the mass point $e_{x}$ at *T* frames is represented in Equation (19).

(19) $$\begin{array}{c} V_{v_{x}}=\sqrt{{\left(v_{x}-v_{x-1}\right)}^{2}} \end{array}$$

$v_{x}$—mass point $e_{x}$ space coordinates at frame *T*;

$v_{x-1}$—mass point $e_{x}$ space coordinates at frame T−1.

**Figure 10:**
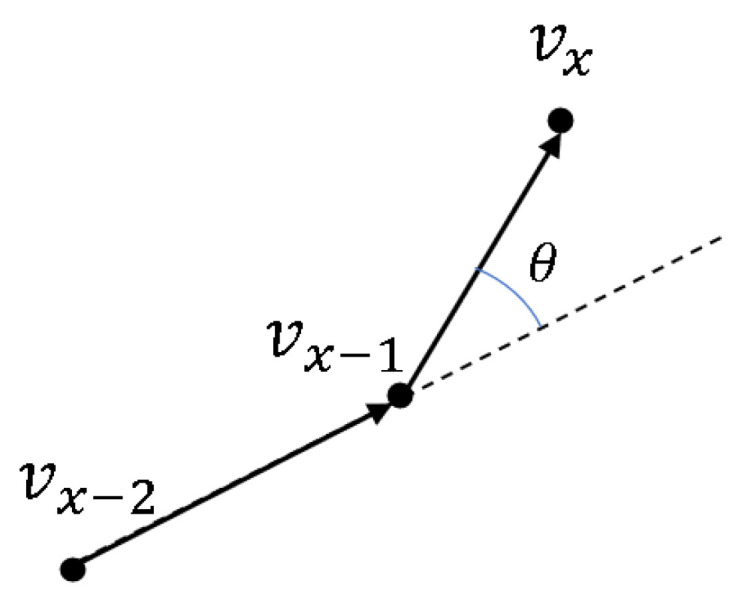
Mass $e_{x}$ angle schematic at frame *T*.

Define the space coordinates at T−2, T−1, and *T* frames as $v_{x-2}$, $v_{x-1}$, $v_{x}$, the angle of the mass point at *T* frames is the angle between the vector $\underset{v_{x-2}v_{x-1}}{\to }$ and $\underset{v_{x-1}v_{x}}{\to }$, as shown in Figure 10. The cosine function of this angle is expressed as Equation (20). In the experiment part, the angle of frame 1 and frame 2 is generally set to 0.

(20) $$\begin{array}{c} \cos\theta =\frac{\vec{v_{x-2}v_{x-1}}.\vec{v_{x-1}v_{x}}}{|\vec{v_{x-2}v_{x-1}}\left|\right|\vec{v_{x-1}v_{x}|}.} \end{array}$$

In addition, we need to calculate the absolute velocities of $e_{A}$, $e_{B}$, $e_{C}$, $e_{D}$, and $e_{E}$; their computation is comparable to the $e_{x}$ velocity calculation. Once all the above data are calculated, we track the trajectory of $e_{x}$ and extract its position and motion characteristics to make a prediction of the next trajectory. Our inputs include the position information and absolute velocity of $e_{A}$, $e_{B}$, $e_{C}$, $e_{D}$, and $e_{E}$ and the angle change of $e_{x}$.

### 6.2. TPT Modeling Architecture

**Figure 11:**
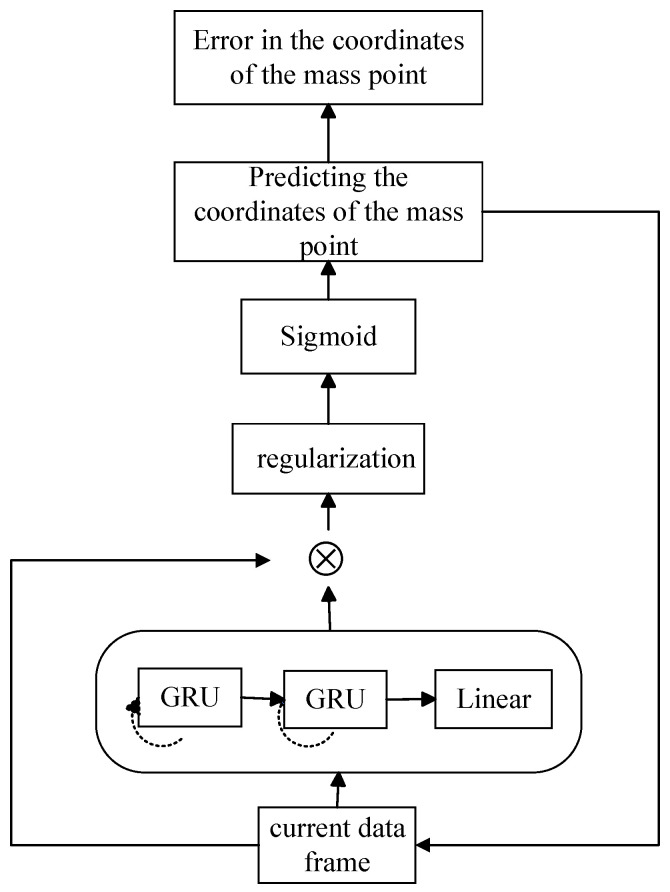
TPT model architecture diagram.

The TPT model is autoregressive, the model predicts the future frames’ trajectory by taking as inputs the previous cyclic state as well as features describing the earlier trajectory at each time step. The entire model forecasts the trajectory’s state in the upcoming *K* frames using the current frame data as input. The TPT network model consists of two GRU layers, each containing 1000 hidden units and a linear activation function Linear. Mul denotes the product of the two matrices. The purpose of regularization and normalization is to avoid overfitting the model and reduce the algorithm generalization error. Finally, after the Sigmoid activation, the mass points prediction coordinates are generated (Figure 11).

## 7. Experiment and Results

The experiments were conducted on a Windows 10 system with an Intel(R) xeon(R) E5-2640 v4 @ 2.40 GHz processor with 32G RAM and the graphics card was an NVIDIA GeForce RTX 2080Ti. The codes were written in Python 3.7, and the entire training and testing were conducted on the PyCharm.

### 7.1. Datasets

The experiment had three parts. Firstly, we compared the LSFE model and RNN model time complexity and accuracy. To confirm the reasonableness of the *L*-MSFCF model, the All-MSFCF model and 2s-AGCN were compared. Lastly, we compared the TPT model with other models regarding the number of parameters and the final average loss error prediction.

The datasets we chose in this experiment were the human3.6m dataset [30], the UCF-Crime dataset [31], and the ShanghaiTech Campus dataset [32], as detailed in Section 3.2. Based on these datasets, we organized eight categories: walking, running, stooping, fighting, vicious kicking, climbing over walls, throwing suspicious objects, and slashing devices. Each video is between 0 and 10 s and the video format is avi. The entire dataset contains 3146 videos and we selected 314 videos as the test set and the rest as the training set.

### 7.2. LSFE Model Test

**Figure 12:**
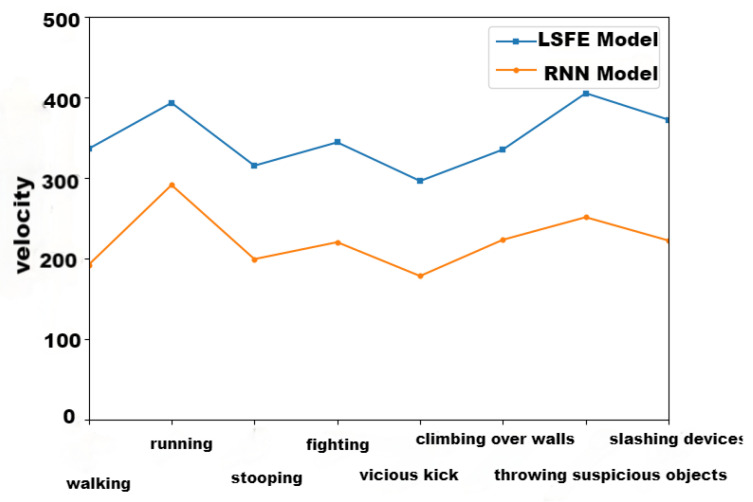
Time complexity comparison of RNN model and LSFE model.

The purpose of lightweight skeleton nodes was to increase the action recognition speed, so we compared time complexity, as shown in Figure 12. Taking 15 frames as a recognition unit, the outcomes supported that the LSFE model’s time complexity was less than the RNN model’s [34]. The results show that the time complexity of LSFE is significantly better than that of the RNN model, and the average recognition speed is about 86.5% higher.

**Figure 13:**
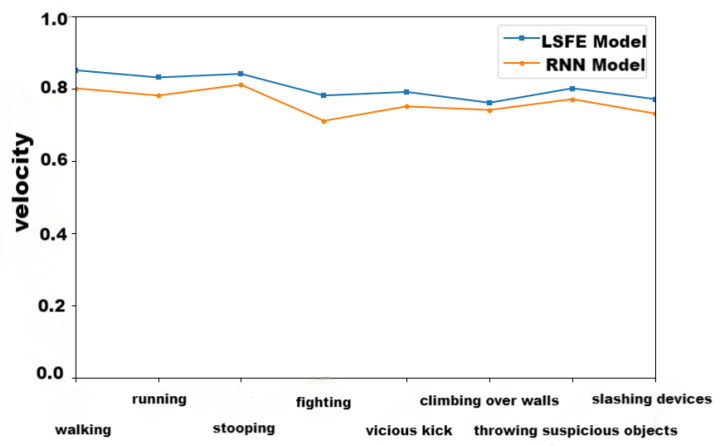
Accuracy rate comparison of RNN model and LSFE model.

In order to further validate the effectiveness of the feature skeleton nodes, this paper compares the recognition accuracy rate between the LSFE model and the RNN model (Figure 13).

The results show that the accuracy of the RNN model is higher than that of the LSFE model for all eight actions. In Table 4, we compared the average accuracy and time in detail. Although the average accuracy of the LSFE model is 4.5% lower than that of the RNN model, its average recognition speed is 86.5% higher. Thus, there was merit in abnormal behavior recognition based on lightweight skeleton nodes.

### 7.3. L-MSFCF Model Test

**Figure 14:**
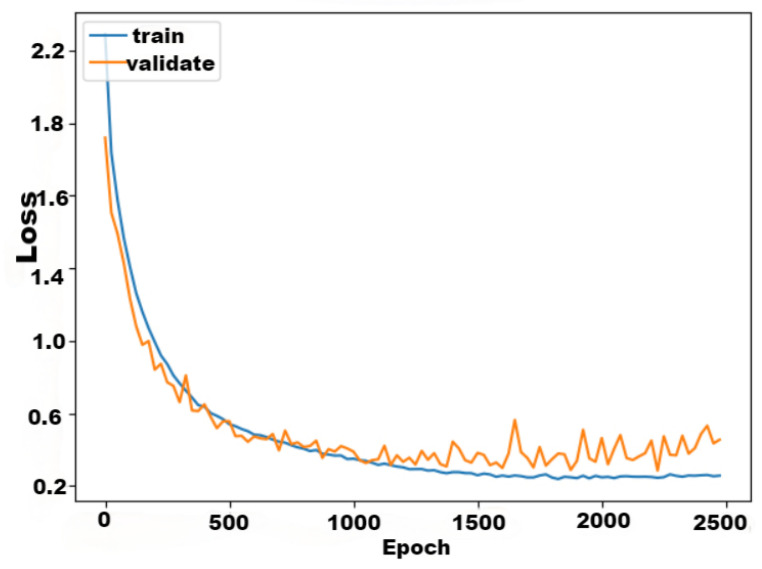
*L*-MSFCF model training iterations versus loss function.

**Figure 15:**
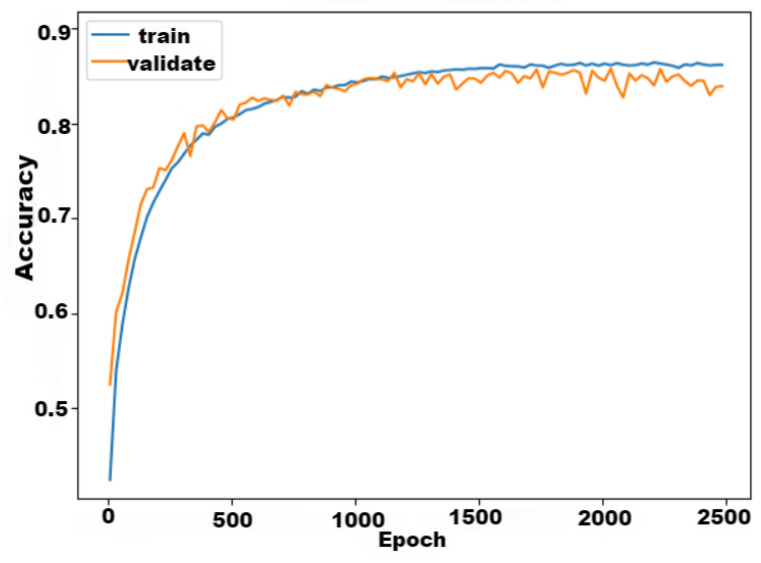
*L*-MSFCF model training iterations versus accuracy.

The *L*-MSFCF model had a good performance in accuracy and loss value after training, the loss rate of the *L*-MSFCF model is displayed in Figure 14, and the accuracy rate is shown in Figure 15.

From Figure 14, the initial loss of the *L*-MSFCF model was as high as around 2.2 during the neural network’s training phase. However, it decreased rapidly after 500 iterations, and the convergence gradually slowed down after about 500 iterations, and the final loss value reached a smaller value. It indicated that the *L*-MSFCF model had a good learning effect.

**Figure 16:**
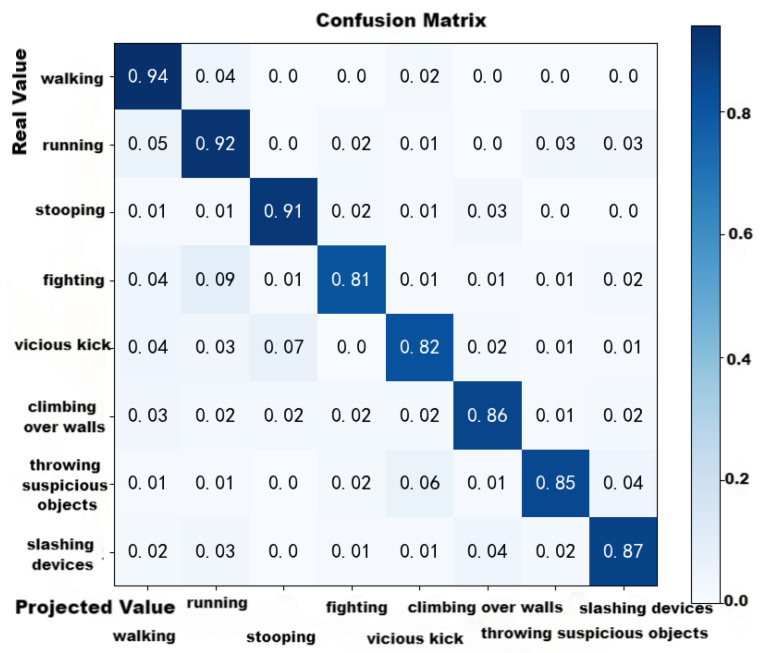
Confusion matrix diagram.

From Figure 15, in the first 1000 iterations, the accuracy curve of *L*-MSFCF converged rapidly, and then after 1500 iterations the accuracy basically stayed stable at a value of about 0.84 (Figure 16).

In addition, we assessed the algorithm’s performance utilizing a confusion matrix. The vertical coordinate represented the real value, whereas the horizontal coordinate indicated the projected value. The diagonal element was the percentage of the projected value to the true value.

The confusion matrix diagram demonstrated that running and walking had higher similarity among normal behaviors, probably because of the higher similarity of the lightweight skeleton nodes in their recognition process. Meanwhile, fighting and running had high similarities in abnormal behavior. Overall, normal behavior was more accurately recognized than abnormal behavior, and the recognition accuracy is 87.3%, so the overall recognition effect basically meets the expectation.

The *L*-MSFCF model took lightweight skeleton nodes as inputs; we regarded the model that had the same network architecture but took full skeleton nodes as the All-MSFCF model.

We compared the *L*-MSFCF model, the All-MSFCF model, the 2s-AGCN model [33], and the LSFE model. Table 5 presented the findings. The results demonstrated that when we input lightweight skeleton nodes, the *L*-MSFCF model’s recognition accuracy outperformed both the 2s-AGCN model and the LSFE model by a large margin. However, the *L*-MSFCF model’s accuracy was lower than that of the All-MSFCF models.

We also compared the time complexity of the *L*-MSFCF model, All-MSFCF model, and 2s-AGCN model to further assess the feasibility of the *L*-MSFCF model (Table 6).

Taking 15 frames as a recognition unit, the *L*-MSFCF model recognition speed was clearly higher than that of the All-MSFCF model and the 2s-AGCN network model. Compared with the All-MSFCF model, the average recognition speed was more than two times higher. Compared with the 2s-AGCN model, the average recognition speed is about 62.7% higher.

The *L*-MSFCF model was much more efficient than the 2s-AGCN model, superior to the 2s-AGCN network model in both recognition speed and accuracy. Even though the *L*-MSFCF model’s accuracy was 5.4% less than the All-MSFCF model’s, the recognition speed was improved by nearly one time. This showed that the *L*-MSFCF model had merit.

**Figure 17:**
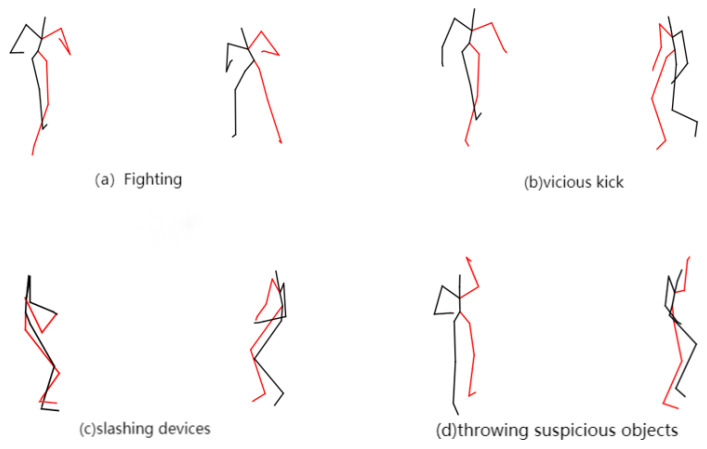
Abnormal behavior recognition effect.

Finally, the method of this paper demonstrates the effect of recognizing some abnormal behaviors. The line in Figure 17 indicates the skeleton outline of the body in each frame. From Figure 17, it can be seen that the method can identify abnormal behavior more accurately.

### 7.4. TPT Model Test

We contrasted the TPT model with the PIF [35] and the *S*-GAN-*P* [36] model in order to emphasize its superiority. Each of them set up the training parameters, trained on the same datasets, predicted the trajectories in the future 15 frames, 30 frames, and 45 frames, and took the final average loss errors as the evaluation indexes. The experiment results are displayed in Table 7.

The findings demonstrated that the TPT model’s predictions for the next 15 and 30 frames outperform those of the other two network models, but as time goes on, the gap between the errors of the TPT model and *S*-GAN-*P* model gradually decreases, and after 45 frames the error of *S*-GAN-*P* model is smaller than the TPT model. It shows that TPT has an advantage in short-term prediction, but its advantage gradually decreases with the increase of time, so the later research can focus on the following long-time prediction.

The *S*-GAN-*P* model has 46.3k parameters and is the smallest model. However, the TPT model only has 17.6k parameters, which is about one-third of the parameters in *S*-GAN-*P*. Regarding inference speed, the quickest approach was *S*-GAN-*P*, which takes 0.0968 s for each inference step. The TPT model has an inference time of 0.0235 s for each inference step, four times faster than the *S*-GAN-*P* model. Table 8 shows that the TPT model has a considerable advantage both in parameter numbers and prediction time. We chose lightweight skeleton nodes and a redesigned convolutional architecture nicely circumvented the problem of substantial data and the usage of a cyclic architecture.

Finally, this paper shows part of the visualization results based on trajectory prediction tracking. In order to make the visualization results clear, we process the mass point $e_{x}$ in the visualization by keeping the X-axis and Y-axis coordinates unchanged, but the Z-axis coordinates are subtracted by half of the height of the body.

**Figure 18:**
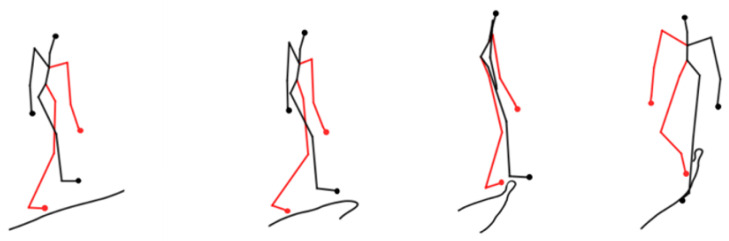
Walking posture prediction visualization results.

**Figure 19:**
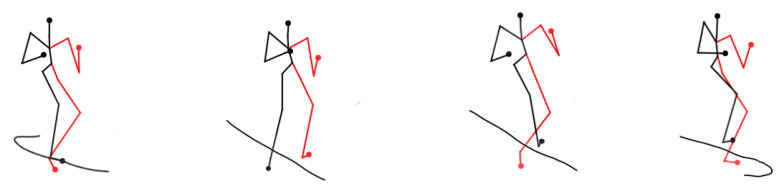
Fighting posture prediction visualization results.

Firstly, Figure 18 and Figure 19 provide the visualization graphs of trajectory prediction results for some of the intact skeleton nodes. In the complete skeleton nodes, we utilize 4, 10, 7, 13, and 15, which represent the right hand, right foot, left hand, left foot, and head as the basis points to calculate the mass points. Figure 18 shows the walking posture prediction visualization results. Figure 19 shows the fighting posture prediction visualization results.

**Figure 20:**
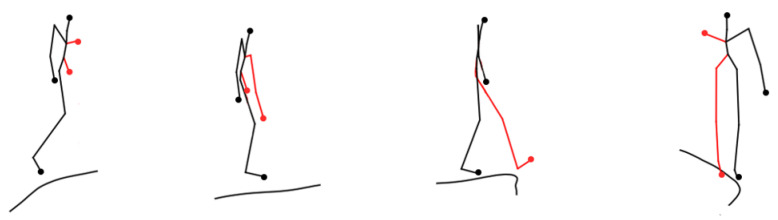
Walking posture trajectory prediction visualization results with occluded skeleton nodes.

**Figure 21:**
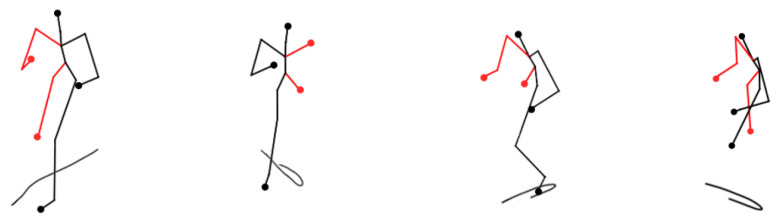
Fighting posture trajectory prediction visualization results with occluded skeleton nodes.

Secondly, Figure 20 and Figure 21 provide the visualization graphs of trajectory prediction results for some of the obscured skeleton nodes. In Figure 20, due to occlusion or other problems, it lacks skeleton nodes 6, 7, 12, and 13, which represent left elbow, left hand, left knee, and left foot. According to the Top order, the left shoulder and left hip are selected as one of the five bases. The other occluded skeleton information in Figure 20 and Figure 21 are sequentially calculated according to the five-bit skeleton screening method.

## 8. Discussion

This experiment verifies that the lightweight skeleton nodes process efficiently increases the timeliness of video action recognition. The LSFE method has a huge advantage in time complexity. The *L*-MSFCF model improves abnormal behavior recognition accuracy by predicting occluded skeletons and using feature fusion. We propose a method based on the previous frame for predicting skeleton data, which reduces the noise generated by the current skeleton data prediction from the distant skeleton data.

In this paper, 15 frames were selected as the most appropriate length of the action sequence. The advantages include a smaller amount of data and less noise, which enables a more accurate capture of video abnormal behaviors. Thus, the TPT model has the highest prediction accuracy under 15 frames, indicating that it is most effective in short-term prediction. The model can predict the trajectory of abnormal behaviors efficiently and quickly, showing its significant advantages in real-time applications in the computer vision field.

In terms of video abnormal behavior recognition and tracking, the research in this paper has achieved some milestones, but the applicability and reliability of the method in complex scenarios have not yet been fully discussed, and the stability of the algorithm’s performance in multiple datasets or scenarios, as well as the applicability of the algorithm to different numbers of skeleton nodes, have not been fully discussed. This paper only studies the video without combining audio, sensors, and other multiple data sources for comprehensive analysis. Future research should focus on the algorithm’s real-time, multimodal fusion and interpretability.

## 9. Conclusions

In this paper, we addressed the problem of a large number of skeleton nodes as well as behavioral occlusion between individuals degrading the abnormal behavior recognition speed and accuracy. We proposed a lightweight multi-stream feature cross-fusion (*L*-MSFCF) model. The model adopted lightweight skeleton node computation, which significantly improved the recognition speed; at the same time, it improved the recognition accuracy by predicting the occluded skeleton nodes and effectively coped with the behavioral occlusion problem. Experiments show that our model achieves an average accuracy of 87.3% for abnormal behavior recognition. In addition, we also proposed the Trajectory Prediction Tracking (TPT) model, which can predict the movement position in real time based on core skeleton nodes, and its short-term prediction average loss error is small. In conclusion, our research effectively solves the behavioral occlusion problem while improving the recognition speed and accuracy, providing new ideas and methods for the development of the video action recognition field. The proposed model is expected to be applied to various practical applications in the fields of security and surveillance.

## Figures and Tables

**Table 1:** Behavior classification.

Identifiers	Action Type	Category Definition	Behaviors
N	Normal behavior	When behavior is consistent with usual behavior, it is quite normal for an individual.	Walking, running, stooping.
A	Abnormal behavior	Can be divided into two kinds: one is the disturbance of order in public places, and the other refers to criminal acts.	Fighting, vicious kicking, climbing over walls, throwing suspicious objects, and slashing devices.

**Table 2:** Lightweight feature skeleton node extraction results.

Action Type	Lightweight Feature Skeleton Nodes
Walking	3, 4, 6, 7, 9, 10, 12, 13
Running	3, 4, 6, 7, 9, 10, 12, 13
Stooping	0, 1, 2, 3, 4, 5, 6, 7, 14
Fighting	3, 4, 6, 7, 9, 10, 12, 13
Vicious kicking	2, 3, 4, 5, 6, 7
Climbing over walls	3, 4, 6, 7, 8, 9, 10, 11, 12, 13
Throwing suspicious objects	3, 4, 6, 7, 10, 13
Slashing devices	2, 3, 4, 5, 6, 7, 10, 13, 0

**Table 3:** Skeleton node sequences for each partition.

Area	Skeleton Node Sequences
A	0, 1, 14, 15
B	4, 3, 2
C	7, 6, 5
D	10, 9, 8
E	13, 12, 11

**Table 4:** Model prediction accuracy and time comparison.

Model	Average Accuracy	Average Time
RNN Model	80.7%	362 ms
LSFE Model	76.2%	194 ms

**Table 5:** Behavior recognition rate accuracy comparison.

	Models	*L*-*MSFCF*	*All*-*MSFCF*	*2s*-*AGCN*
Behaviors				
Walking		0.94	0.96	0.87
Running		0.92	0.95	0.88
Stooping		0.91	0.93	0.84
Fighting		0.81	0.87	0.73
Vicious kicking		0.82	0.89	0.78
Climbing over walls		0.86	0.95	0.83
Throwing suspicious objects		0.85	0.94	0.83
Slashing devices		0.87	0.93	0.84
Average accuracy		0.873	0.927	0.825

**Table 6:** Time- consuming comparison.

Models	Parameter Numbers	Average Recognition Time
All-MSFCF	377.3 k	347 ms
2s-AGCN	117.9 k	258 ms
*L*-MSFCF	62.6 k	162 ms

**Table 7:** The model’s average loss errors in each frame prediction.

Model	15 Frame Errors	30 Frame Errors	45 Frame Errors
PIF	0.33	0.43	0.65
*S*-GAN-*P*	0.22	0.35	0.51
TPT	0.19	0.32	0.57

**Table 8:** Model prediction times comparison.

Model	Parameter Number	Prediction Time
PIF	360.3k	132 ms
*S*-GAN-*P*	46.3k	97 ms
TPT	17.6 k	23 ms

## Data Availability

A publicly available dataset was analyzed in this study. The Human3.6m dataset can be found here: http://vision.imar.ro/human3.6m/description.php (accessed on 18 August 2018). Dataset UCF-Crime can be found here: https://www.dropbox.com/sh/75v5ehq4cdg5g5g/AABvnJSwZI7zXb8_myBA0CLHa?dl=0 (accessed on 18 September 2018). Dataset ShanghaiTech Campus can be found here: https://svip-lab.github.io/dataset/campus_dataset.html (accessed on 20 April 2022).
